# How to Distinguish Oleomargarine

**Published:** 1880-12

**Authors:** 


### HOW TO DISTINGUISH OLEOMARGARINE.

According to the Philadelphia "Medical Times," Dr. G. C. Wittstein, in an Austrian pharmaceutical journal, explains how to distinguish cream butter from "ox butter" by means of the microscope. Place a small piece of the butter npon an object-glass, spread it out by means of a cover-glass, and observe it under a power of 300 to 400. If it is pure butter the whole field is filled with extremely fine globules, which are entirely destitute of any approach to crystalline form. If the butter is artificial, or a mixture of both, the field presents numerous angular or acicular particles between the globules. These crystalline particle are derived, no doubt, from the stearine which forms part of the beef tallow in artificial butter. Lard does not show any such crystalline particles.

